# Outdoor Inclined Plastic Column Photobioreactor: Growth, and Biochemicals Response of *Arthrospira platensis* Culture on Daily Solar Irradiance in a Tropical Place

**DOI:** 10.3390/metabo12121199

**Published:** 2022-11-30

**Authors:** Tjandra Chrismadha, Awalina Satya, Ika Atman Satya, Rosidah Rosidah, Azalea Dyah Maysarah Satya, Ratih Pangestuti, Ardiyan Harimawan, Tjandra Setiadi, Kit Wayne Chew, Pau Loke Show

**Affiliations:** 1 Research Center for Limnology and Water Resources, National Research and Innovation Agency (BRIN), KST Soekarno, Jl. Raya Bogor-Jakarta km46, Cibinong, Bogor 16911, Indonesia; 2 Research Center for Computation, National Research and Innovation Agency (BRIN), Komplek Cisitu, Jl. Sangkuriang, Coblong, Bandung 40135, Indonesia; 3 Department of Chemical and Environmental Engineering, Faculty of Science and Engineering, University of Nottingham Malaysia, Jalan Broga, Semenyih 43500, Selangor Darul Ehsan, Malaysia; 4 Research Center for Food Technology and Processing (PR TPP), National Research and Innovation Agency (BRIN), Yogyakarta 55861, Indonesia; 5 Department of Chemical Engineering, Institut Teknologi Bandung, Jl. Ganesa No 10, Bandung 40132, Indonesia; 6 School of Chemistry, Chemical Engineering and Biotechnology, Nanyang Technological University, 62 Nanyang Drive, Singapore 637459, Singapore; 7 Zhejiang Provincial Key Laboratory for Subtropical Water Environment and Marine Biological Resources Protection, Wenzhou University, Wenzhou 325035, China; 8 Department of Sustainable Engineering, Saveetha School of Engineering, SIMATS, Chennai 602105, India

**Keywords:** cyanobacterium, *Arthrospira platensis*, tropical areas, plastic column photobioreactors, phycocyanin, irradiance, outdoor culture

## Abstract

Implementation of outdoor photobioreactors has been challenged by an extremely oversaturated daily peak of solar irradiance. This study aims to understand the role of column size and paranet shading as well as to investigate the most convenient light control in outdoor cyanobacterial culture. The photobioreactor (PBR) consisted of plastic columns with a diameter of 12.74 cm (PBRd-20) and 31.85 cm (PBRd-50) laid outdoors and inclined at 158.22° upwards against solar radiation, while paranet shading was provided at 0%, 50%, 70%, and 90% shading capacity. A semi-continuous culture of cyanobacterium *Arthrospira (Spirulina) platensis* was conducted for 6 weeks with weekly monitoring of the growth parameter as well as the proximate and pigments content, while the daily irradiance and culture maximum temperature were recorded. The result shows that the column diameter of 12.74 cm had a lethal risk of 44.7% and this decreased to 10.5% by widening the column diameter to 31.85 cm. This lethal risk can be eliminated by the application of a paranet at a 50% reduction level for the column diameter of 31.85 cm and a 70% reduction level for the column diameter of 12.74 cm. The highest culture productivity of 149.03 mg/(L·day) was achieved with a PBRd-20 with 50% shading treatment, but a PBRd-50 with 90% shading treatment led to an increase in the protein and phycocyanin content by 66.7% and 14.91%, respectively.

## 1. Introduction

Microalgae and cyanobacteria are potential raw materials for various industrial sectors, including foods, feeds, biofuels, organic products, bioplastics, pigments, cosmetics, pharmaceuticals, and biosorbents [1,2,3,4]. On the other hand, sustainable microalgal and cyanobacterial production is still facing various obstacles that must be overcome [5,6], among which is the choice of an effective culture system, particularly in outdoor environments [7]. Outdoor microalgal and cyanobacterial biomass production has been operating for decades, mostly employing open ponds, particularly due to simple technology and lower investment costs [8,9]. This cultural practice, however, is difficult to control during their operation and is susceptible to contamination [10]. Accordingly, there have been suggestions originating from a long time ago to employ a closed culture system, which is commonly named a photobioreactor (PBR), for producing microalgae and cyanobacteria biomass. The PBR is known to have better control of operating conditions, making the culture reproducible and possessing fewer contamination problems [8,11]. There have been some reports mentioning the development of PBR for culturing various cyanobacteria and microalgae, such as *Spirulina (Arthospira) platensis, Phaeodactylum, Nannochloropsis, Chlorella*, etc. [12,13,14]. PBR has also been shown to provide good manufacturing practice requirements for food, feed, and additives [15]. However, there are some suggestions for further development efforts, emphasizing the need for better design and operational means to comply with the optimal conditions required for specific microalgae growth, along with the cost feasibility [12].

One major concern in PBR development is light availability and utilization. The tropical area, which only has two seasons, has been noted as an ideal place for PBR implementation to produce microalgae and cyanobacteria biomass since solar irradiance is available all year long at relatively high quantities [16]. In fact, outdoor PBR implementation is mainly hindered by a high fluctuation of daily solar irradiance that culminates to more than 100,000 lux (819.7 W/m^2^), which leads to photorespiration and eventually kills the cultured microalgae cells. At the same time, the placement of a growth medium within a closed transparent column, that may eliminate the evaporation problem [17], has the disadvantage of heat accumulation when sunshine is scorching, also hindering the algal culture sustainability. An effective approach, particularly in terms of PBR design, is still needed to bring it into large-scale implementation in tropical areas.

Measures to understand light response behavior among organisms, including microalgae and cyanobacteria, have been carried out since the beginning of scientific development [18]. The light response curves have been well revealed, showing saturation points, which are the levels of light intensity that can be used effectively by photoautotrophic organisms, mostly below 20,000 lux (163.9 W/m^2^). However, how organisms cope with fluctuating irradiance remains unclear. In addition, besides daily oscillation due to day-night cycles, light fluctuation is also complicated by the weather, making efforts to manage solar irradiance for optimal photosynthetic purposes more difficult. In the case of algal and cyanobacterial culture development, there has been an agreement among some experts that cultured cells adapt better to the average irradiance input [19]. However, as long as algal culture safety concerns are addressed, the sporadic peak irradiance level, which frequently reaches up to 100,000 lux (819.7 W/m^2^) or more, should not be neglected. Measures to give protection to avoid photoinhibition on the cultured cells must be undertaken, as was done in this study by utilizing the paranet roofing. A paranet is a type of net made from plaited mats of polyethylene threads that is widely used for shading purposes in agricultural fields.

The physiological states of both microalgal and cyanobacterial cultures associated with irradiance are also determined by the culture’s volume. There have been some reports demonstrating that more efficient irradiance usage for biomass production could be obtained by adjusting the ratio of surface to volume (S/V), which can also be interpreted to mean that the microalgal culture vulnerability related to excessive irradiance is determined by this S/V factor [20]. It means that the higher photosynthetic rate obtained by any S/V values will be followed by increasing susceptibility to oversaturated irradiances, so it needs very careful attention [21]. In addition to inducing photorespiration, high levels of light intensity leads to heat accumulation in the algal culture, which can also be very harmful to the algal growth. In terms of photorespiration, the extent of heat accumulation susceptibility is also determined by the S/V values, as the wider culture surface receives more light, while the heat buffer capacity at the same volume is relatively constant. Heat accumulation problems in outdoor algal culture have been widely reported [22] and various efforts to overcome them, such as the application of heat exchangers, are still considered very expensive [23]. In this study, it is proposed that applying paranet roofing will enhance more efficient natural irradiance control, particularly in efforts to sustain outdoor algal cultures against the extremely high natural irradiance of particular tropical areas. The rationale for this selection is mainly that the paranet material is commonly available in many agricultural shops at various light reduction levels [24], so it would be ready for application anywhere and anytime. The material is also strong enough to be used in direct sunlight and has been shown to protect plants that are sensitive to light.

It must be underlined that the above mentioned purpose of the paranet roofing application is mainly to protect algal and cyanobacterial cultures from extremely high irradiance in day peak times and must take into account the consequences of daylight reduction, which might lower the potential algal and cyanobacterial photosynthesis. The ideal shading level should be set at the point where the algal and cyanobacterial cells can survive and recover their normal metabolism soon after the damaging condition is over, and it must also consider the purpose and target products of those cultures. Based on this idea, this study was conducted to find out how shading affects the cyanobacterial metabolisms and to figure out the best way to control light for growing cyanobacterium in more stable outdoor environments.

## 2. Experimental Design

### 2.1. Arthrospira (Spirulina) Platensis and Culture Media

*A. platensis* was obtained from the culture collection of the Research Center for Limnology and Water Resources—the Indonesian National Research and Innovation Agency, Soekarno Science and Technology Center, Indonesia (in Indonesian called KST Soekarno, BRIN), which is located on S 6°29′31.115″; E 106°51′11.589″. The culture medium used was the modified Zarrouk medium [25], in which the modification was accomplished by lowering the NaHCO_3_ concentration down to 12 g/L and KNO_3_ to 1 g/L [25]. The chemicals were of technical grade except for the trace element solutions. The stock culture was maintained in the photoautotroph-grown mode in a controlled environment with light provided by two white fluorescence lamps (Philips 18W, Holland) placed on one side of the culture. This experiment proceeded in a monoculture condition, using filtered tap water (through a 0.1µm membrane apparatus) as a solvent for medium preparation.

### 2.2. Outdoor PBR Experiment

**Figure 1:**
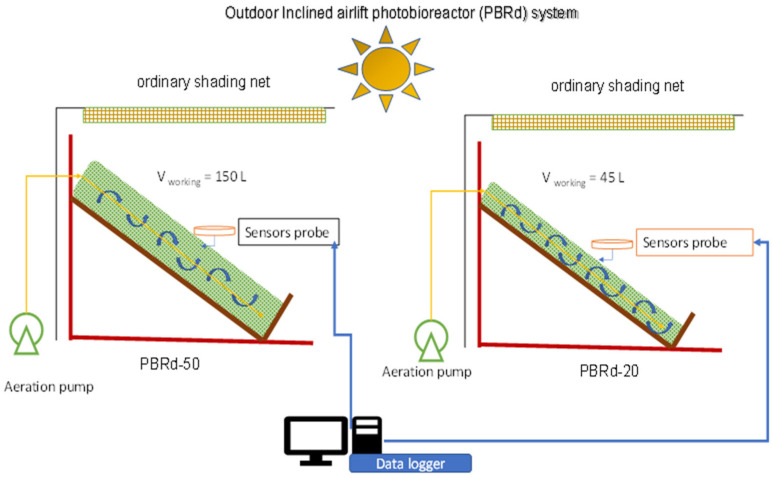
Schematic diagram of an outdoor inclined plastic column photobioreactor PBRd2021 series. The diameter of the plastic column (which contains *A. platensis* broth) varied between 12.74 (right) cm and 31.85 cm (left), giving the names PBRd-20 and PBRd-50.

**Figure 2:**
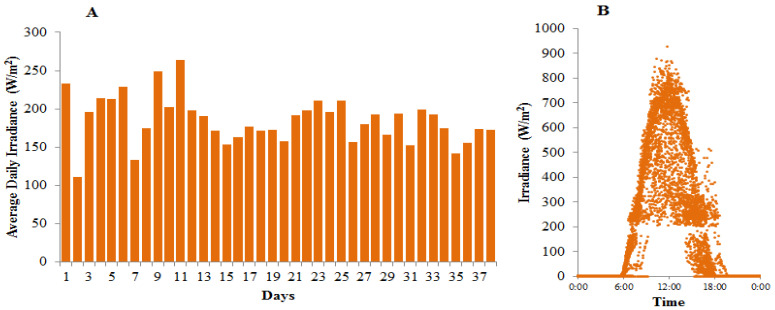
Daily averages (**A**) and oscillations (**B**) of aerial solar irradiance during observation time.

The photobioreactor (PBR) was constructed from a plastic column (Polyethylene, PE 80 µm thick) with two different widths, which were 20 cm (PBRd-20) and 50 cm (PBRd-50). It gave the column a diameter of 12.74 cm and 31.85 cm, and provided a working volume of 45 L and 150 L respectively when fully filled with culture broth. The plastic column was laid on wooden racks adjusted to an inclination of 158.22° upwards against solar radiation (Figure 1). The PBR was placed outdoors on the rooftop of our laboratory building and received daily illumination from around 06:00 to 18:00 (Figure 2). The supporting rack was composed of multiplex wood, put on a wooden frame, and covered with an ebonite sheet. An aeration pipe was put into the bottom of the culture column and connected to an aerator pump (Amara Aa-666-China) to provide a continuous bubbling of natural air (with atmospheric CO_2_ content) that created turbulence mixing to keep cells from clumping and to allow a good spread of light for the photosynthesis process.

A semi-continuous culture system was employed during this experiment, utilizing a weekly harvest of as much as 2/3 of the culture volume and replacing it with an appropriate newly fresh medium. It was designed to avoid medium nutrient exhaustion, which might interfere with the treatment observation. The culture ran for 6 weeks, commencing on 9 September 2021, and ending on 21 October 2021. Samples were collected from the weekly harvested culture in order to measure biomass (culture) density as well as biochemical (protein, carbohydrate, phycocyanin, and chlorophyll-a) analysis.

Shading treatment was conducted (in duplicate) using an ordinary paranet (Dragon-Paranet, Indonesia) which had the light reduction varied from 50%, 70%, and 90%, denoted as I = 50%, I = 30%, and I = 10%, and a control treatment (without shading), which was denoted as I = 100%. The treatment was done under the assumption that an outdoor algal culture needs protection from extreme solar irradiation to make it sustainable. Different levels of light reduction were also made to widen the range of daily irradiance down to the surface of the culture. This made it possible to evaluate the effect of outdoor irradiance on algal culture performance in a more thorough way.

The daily solar irradiance was measured indirectly using a lux meter-logger (LX113S, Lutron Taiwan) mounted next to the PBRs site, and the obtained illuminance data was converted to irradiance (expressed in W/m^2^) following a method developed by Michael et al. [26]. It continuously recorded the direct exposure of sunlight on the rooftop area, while the irradiance values beneath the shading treatment were obtained by multiplying the direct exposure values with the paranet light penetration level. The light recording was set for every minute all day long. The compiled data was then calculated for the average daily solar irradiance. The daily maximum temperatures were measured with eight thermometers of maximum and minimum temperatures (HTC-2, China) that were put on each of the eight plastic tubes based on how they were shaded and the diameter of the plastic tubes. The temperature probes were inserted underneath the plastic column to avoid direct exposure to sunlight that might cause thermal accumulation to bias the measurement. The max/min temperature was recorded, but this paper only discussed the obtained maximum temperature data.

## 3. Procedure

Regression-correlation analysis was performed on Microsoft^®^ Excel^®^ MSO2021 and SigmaPlot14 (Systat Software Inc., San Jose, CA, USA) to demonstrate algal culture response models in terms of maximum temperature to daily average irradiances, with these daily irradiances being added to the weekly average to assess its influence on *A. platensis* growth performance and biochemical metabolism. The cyanobacterium culture performance was evaluated in terms of culture density (CD), specific growth rate (SGR), biomass productivity (P), and biochemical properties including proximate content (protein, carbohydrate, lipid, and ash) as well as pigments: chlorophyll and phycocyanin. The measurement was carried out weekly according to the regular harvesting time for 6 weeks.

Biomass was expressed in terms of dry weight (DW), which was obtained gravimetrically from aliquot samples as much as 500 mL for the light culture and 250 mL for the thick culture, filtered through pre-weighted Whatman GF/C filter paper, and dried at room temperature before being oven-dried at 105 °C for 2 h to determine the dry weight (DW), and then furnaced at 600 °C for another 2 h to determine the ash content. The DW was determined by the weight subtraction of filter papers, which was the biomass sample after the oven with the empty ones, while its subtraction with the furnaced filtered sample was applied to calculate the ash content. The information was then used to assess culture performance in terms of culture density (CD; g/L), specific growth rate (SGR; %/day), and biomass productivity (P_x_; mg/(L·day)). Calculation of SGR was conducted as follows (Equation (1)).

(1) $$\mu_{\max}=\frac{\left(\ln N_{2}-\ln N_{1}\right)}{\left(t_{2}-t_{1}\right)}\times 100$$

where N_1_ and N_2_ were concentration of cells at the beginning of the observation time (t_1_) and at the end of the observation time (t_2_) of the exponential growth. Meanwhile, P_x_ (g/(L·day)) calculation was conducted according to Equation (2).

(2) $$P_{X}=\frac{\left(X_{t}-X_{0}\right)}{\left(t_{x}-t_{0}\right)}$$

where X_t_ was the biomass concentration (g/L) at the t cultivation period (t_x_) and X_0_ was the initial biomass concentration (g/L) at t_0_ (day).

The protein content was determined according to Lowry et al. [27] based on bovine serum albumin (BSA) standards, over 10 mL of centrifuged culture aliquot samples. The carbohydrate content determination was also carried out upon 10 mL centrifuged culture aliquot samples according to the colorimetric phenol method using glucose as a standard [28]. The protein and carbohydrate contents of the samples were determined using a spectrophotometer, UV-VIS Thermo Scientific ^TM^ GENESYS 10S (Waltham, MA, USA). The lipid content was analyzed gravimetrically following the methanol: chloroform: water separation method and dried over an N_2_ stream [29].

The time course of phycocyanin was observed by extracting 10 mL of the algal broth sample and quantifying it according to the standard method [30]. For the determination of phycocyanin content, the harvested cells were centrifuged at 6000 rpm (OHAUS model FC5706, USA) for 15 min. The obtained *A. platensis* biomass was rinsed with demineralized water twice. The phycobiliprotein was extracted from the sample using a 0.05 M phosphate buffer through three cycles of freezing and thawing. When the blue color emerged, the sample was then centrifuged at 6000 rpm for 15 min, and the supernatant was collected. The absorbance was read at 615 nm and 652 nm using a spectrophotometer, UV-VIS Thermo Scientifics GENESYS 10S (Waltham, MA, USA). The spectrophotometric method [31] was used to measure the amount of chlorophyll-a (Chl-a) in the harvested biomass of *A. platensis.*

## 4. Results

### 4.1. Irradiance vs. Maximum Temperature

**Figure 3:**
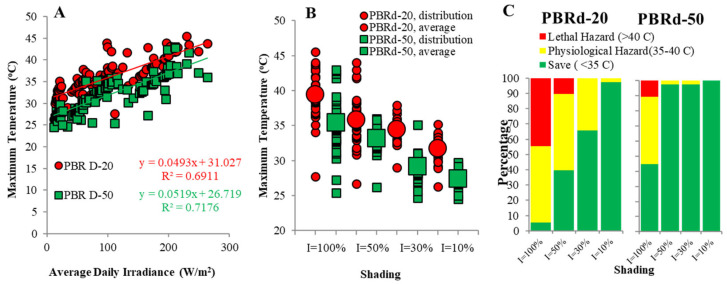
Effect of daily irradiance, paranet shading, and column size on temperatures (**A**,**B**) and hazard level (**C**) of outdoor *A. platensis* culture in a tropical area.

Daily solar irradiance observed during this experiment (13 September–20 October 2021) ranged from 133.69 W/m^2^ to 263.77 W/m^2^, while the highest light intensity recorded was 926.23 W/m^2^ on 20 September 2021 (Figure 2). The maximum temperature of the *A*. *platensis* culture was determined by the above daily irradiances. For this experiment, order 2 polynomial models (Figure 3) can explain this phenomenon.

The thinner column was more sensitive to solar irradiance, as indicated by the higher maximum temperature reaching the same light levels. There were two coincident algal culture crashes in the PBRd-20 that occurred with high daily irradiance levels of 293.27 W/m^2^ which were stimulated by the raising of culture temperature to 45.5 °C, while those in the PBRd-50 survived since they only attained a maximum temperature of 41.0 °C at an irradiance of 298.93 W/m^2^. This observation confirms some previous reports on microalgae and cyanobacteria culture damages attributed to the extreme increase in culture temperature due to uncontrollable natural solar irradiation [32,33]. As shown in Figure 3, in almost 44.7% of this 38 days of experimental time, the *A. platensis* culture suffered from a maximum temperature above 40 °C in the PBRd-20 culture without paranet roofing. This hazard was decreased to 10.5% when the paranet was applied at a 50% light reduction level, and there was no more potential hazard at a 70% light reduction level. When the column diameter was increased to 31.85 cm, the number of days with a high temperature of over 40 °C without a paranet was cut down to 10.53%, and the lethal hazard was eliminated by using a paranet that blocked at least 50% of the incident light.

### 4.2. Growth Performance and Biochemical Content

Table 1 summarizes the weekly baseline assessment of *A. platensis* growth and biochemical content in an outdoor inclined plastic column photobioreactor with different column diameters and light regimes controlled by a paranet application. In this experiment, there was no mechanism to control other growth environments, so the results reflected how the two experimental factors worked together with all the coincidental shaped outdoor conditions.

Both the column diameters and light regimes had a remarkable influence on both the cyanobacterial growth performance and the biochemical content. The thinner column PBR achieved better growth performances in terms of culture density (CD), specific growth rate (SGR), and biomass productivity (P), but the thinner column increased the culture’s vulnerability to the occasional excessive natural solar irradiation, especially when it occurred on two or more consecutive days. As shown in Figure 2, the daily light oscillation shapes up the higher maximum temperature in thinner columns beyond the tolerable point and has an impact on the algal growth performance. It had a consequence of minor growth performance in the PBRd-20 culture without paranet roofing compared to a protected one. Application of the PBRd-20 culture with paranet roofing of 50% shading capacity resulted in the best algal culture growth performance, which corresponds to a maximum productivity value of 149.03 mg/(L·day). The PBRd-50 culture only achieved the maximum productivity of 63.71 mg/(L·day) when applied without shading.

**Figure 4:**
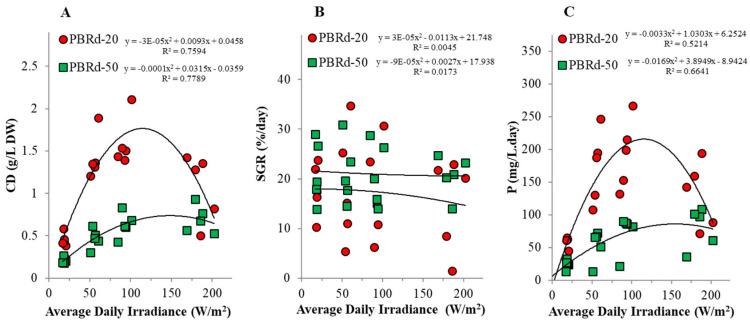
Response of outdoor *A. platensis* culture cell density (**A**), specific growth rate (**B**), and biomass productivity (**C**) to natural daily irradiances in a tropical area.

Regression-correlation analysis revealed some very interesting phenomena, where close relationships were observed between daily irradiances and algal culture density (Figure 4a), as well as biomass productivity (Figure 4c), but not with the specific growth rate (Figure 4b). It indicates that under fluctuated daily irradiances, algal culture has an adaptation mechanism through self-culture density regulation to keep the SGR at a certain rate, which is associated with the most convenient level of algal cell metabolism. The occurrences were consistent in both column sizes with a sharper curve in the thinner column, so as the thinner column achieved higher culture density and productivity, it was more sensitive to environmental light fluctuation.

**Figure 5:**
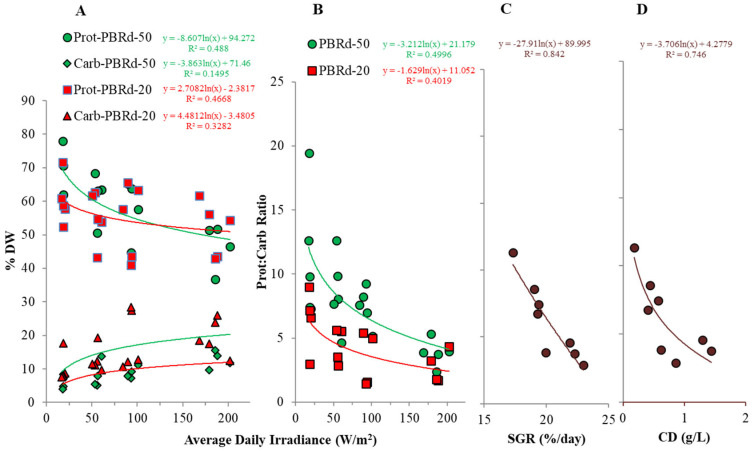
Protein and carbohydrate content of an *A. platensis* outdoor culture as a function of daily irradiance (**A**), and correlation between the protein: carbohydrate ratio and daily irradiance (**B**), specific growth rate (**C**), and culture density (**D**).

Table 1 also shows that the proximate composition of *A. platensis* grown in the outdoor plastic column photobioreactor was dominated by protein, which made up 36.8–78.0% DW, followed by carbohydrate, 2.4–27.5% DW, lipid, 4.2–20.9% DW, and ash, 5.9–20.9% DW. The determination of daily irradiance levels was more pronounced on the algal protein and carbohydrate content. Higher carbohydrate content at the expense of lower protein content consistently occurred with higher daily irradiances, in which the phenomenon was encouraged more by the thinner column sizes. This fact was also observed in terms of the protein/carbohydrate ratio, which tended to be higher in the wider column culture (Figure 5). This observation is consistent with some previous observations that were largely associated with more intensive photosynthesis under conditions of high light intensity uncoupled by the enzymatic processes of protein synthesis, so as the first-order photosynthetic products of simple carbohydrates are accumulated in the cells. This circumstance can also be attributed to the higher maximum temperature in the narrower column culture (Figure 3), as it has been previously reported that sub-optimum temperature leads to cell stress, which reduces protein synthesis associated with functional structures and channels photosynthetic products into storage materials [33], which in *A. platensis* is particularly in the form of carbohydrates.

**Figure 6:**
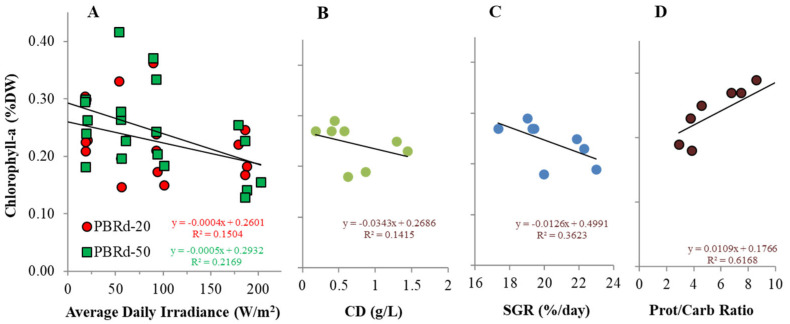
Chlorophyll-a content of an outdoor *A. platensis* culture in response to daily irradiance (**A**), and its relationships with culture density (**B**), specific growth rate (**C**), and protein:carbohydrate ratio (**D**).

**Figure 7:**
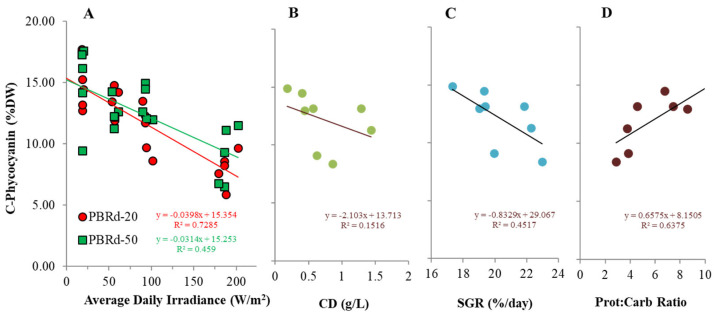
C-phycocyanin content of an outdoor *A. platensis* culture in response to daily irradiance (**A**), and its correlations with culture density (**B**), specific growth rate (**C**), and protein:carbohydrate ratio (**D**).

The chlorophyll content of *A. platensis* in this experiment ranged from 0.15% DW to 0.42% DW, while the phycocyanin content was from 5.9% DW to 17.3% DW (Table 1). In comparison to previous reports [1], these values are within range. There were trends of increasing both the pigment content with lower irradiance, but the correlation values were considerably higher in phycocyanin compared to that in chlorophyll (Figure 6 and Figure 7). A similar phenomenon is obtained when the content of the algal pigment is plotted against the culture density and specific growth rate. Instead, it had remarkably stronger correlations with the protein: carbohydrate ratios, in which higher protein: carbohydrate values tend to increase the algal pigment content.

## 5. Discussion

The existing natural solar irradiance has a tremendous influence on outdoor algal cultures in tropical areas. At noon, the irradiance level experiences an overwhelming culmination far above the level required for algal photosynthesis, and at a certain level, it delivers a fatal impact on the algal culture. This hazard is mostly related to heat accumulation as the culture column receives excessive irradiance during peak illumination time. The extent of heat accumulation is greatly associated with the column size, as the bigger the column, the larger the culture volume, which has an impact on the better temperature buffer capacity [33]. Therefore, it is suggested that in terms of open space column photobioreactor development, the culture resilience against irradiance fluctuation can be arranged by regulating the column size, in which the larger size would enhance better tolerance. A larger column size, however, has widely been known to induce self-shading effects that retard the algal culture growth at sub-saturated light conditions, so compromising column size has to be figured out to encourage the highest level of algal growth at a light level just below the damaged point [34]. It means that the S/V arrangement that has been suggested for culture productivity enhancement also has to be directed to reinforce algal culture resilience toward uncontrollable natural solar irradiation so that the culture productivity can be optimally attained in line with the protection against the hazardous, overwhelming natural light.

This study has also revealed the potential use of paranet for protecting outdoor cyanobacterial culture against extreme natural light, which can be applied in combination with the column size regulation mentioned above. In this case, paranet is employed to provide shading at an optimum level in which cyanobacterial culture productivity and protection against abrupt light become considered. It has been shown that the paranet application can effectively diminish and even eliminate heat accumulation problems related to extremely high natural solar irradiance, such as a 50% shading level at 31.85 cm diameter column size and a 70% shading level at 12.74 cm diameter column size. This finding is consistent with a previous report that showed higher photosynthetic activity of an *A. platensis* outdoor culture with 25% irradiance shading [35] Some reports have emphasized heat accumulation as a major problem in closed photobioreactor systems, particularly in outdoor applications, while using cooling systems to overcome the problem will require significant investment and operational costs [25]. This study reveals a possible way to overcome this problem by means of paranet shading and its combination with the column size. It is also supported by the fact that the material has been massively used in agricultural fields, so it is readily available almost everywhere at a low price, while the installation is also simple and durable under direct sunlight application. The various shading levels commonly available on the market make it easy to meet the requirements for algal culture light control.

There is, of course, a great concern at the level point where the irradiance must be maintained. Although natural light is widely considered an uncontrollable growth factor, the most convenient point must be determined as guidance to achieve an optimum management goal. A large number of studies have been carried out in an effort to understand how algal cultures respond to light conditions, in which besides the intensity level, the light-dark cycle has also become an important issue [36]. Unlike on a laboratory scale where light becomes the limiting growth factor, this study shows an insignificant correlation between daily light level and the algal growth rate, which might be attributed to mostly saturated light conditions. As has been widely known, the algal light response curve divides the algal growth rate according to the light state, and points to a saturation point where algal cells can no longer take advantage of the light intensity increase [37]. However, this experiment reveals that even though natural irradiance above the saturation level does not negatively affect the algal growth rate, it still greatly determines cyanobacterial culture productivity, as shown by a close correlation between light level and algal culture productivity, which can be conveniently explained by utilizing hyperbolic models following second-order polynomial functions (Figure 4). In this saturated light condition, cyanobacterial culture anticipates increasing irradiance by multiplying the culture density to provide a greater number of photosynthetic machines to harvest light and gain a higher biomass productivity level. Therefore, it is considered a self-regulatory mechanism within the cyanobacterial culture to increase the cell density in response to the irradiance increasing above the saturation point to gain more efficient light utilization.

In terms of cyanobacterial culture adaptation to light and dark cycles, this study provides a complementary result to previous reports to suggest that cyanobacterial cultures adapt to regular long-term light-dark cycles in terms of average light intensity. Borowitzka and Vonshak [38] divided the light-dark cycle into three categories. The first is a short light-dark cycle of less than a few seconds, which is generally induced by culture mixing, which is well known as the flashing light effect. The second is a relatively slower light-dark cycle related to diurnal changes in solar irradiation. The third is the long-term cycle as the result of mainly the annual climate variations. While in these first two light-dark cycle regimes, algal and cyanobacterial cells have been recognized to adjust their physiological activities following the average light intensities impinging on the cell surface, the algal adaptation scheme related to seasonal light change has not been well documented [39]. This experiment reveals that even at a weekly period of fluctuating outdoor daily irradiance, algal cultures’ growth performance was largely determined by the average light intensity received by the culture during the culture period. It suggests that the development of outdoor algal culture must consider the local natural solar irradiance property and set a control measure for the optimal amount of light energy for photosynthesis while avoiding the incidence of harmful daylight levels.

Light also has a great influence on the algal *A. platensis* biochemical composition, which is observed in terms of the protein: carbohydrate ratio and indicates the balance state of photosynthesis that produces the first product of carbohydrate and biosynthesis of protein in the cyanobacterial cells. A trend of exponential decrease in the protein: carbohydrate ratio with daily irradiance occurred during this experiment, which can be explained in terms of two phenomena. Firstly, higher light encourages photosynthesis processes that produce carbohydrates, and secondly, the higher irradiance also increases culture temperature, which induces cyanobacterial cell stress and reduces the enzymatic capacity for protein synthesis. In line with this, stronger exponential correlations were also revealed between the protein: carbohydrate ratio and the algal-specific growth rate, and the culture cell density parameters, which notably represent the cyanobacterial growth performance, suggesting the importance of light control at the optimal level related to protein biosynthesis to enhance the algal culture growth and productivity. This is understandable because protein materials are usually synthesized to construct functional organelles, which are very important for cell growth and reproduction.

Light tends to decrease both pigments of chlorophyll and phycocyanin content in cyanobacterial culture, in which the phycocyanin content is more sensitive to light alteration, and the magnitude is larger in thinner columns. Less obvious correlation patterns are obtained when the content of the cyanobacterial pigments is plotted against the culture density and specific growth rate. Instead, it had remarkably stronger correlations with the protein: carbohydrate ratios, in which higher protein: carbohydrate values tend to increase the algal pigment content. This shows that irradiance indirectly determines the cyanobacterial pigment synthesis by shaping the cyanobacterial cell’s internal protein: carbohydrate ratio and is interpreted that protein synthesis is important to support pigment formation in the cells. This point of view is supported by the fact that both chlorophyll and phycocyanin mainly consist of protein materials [40], so the synthesis is largely controlled by the protein availability in the cell. As seen in Figure 5, the protein: carbohydrate ratio tends to decrease with higher irradiance, so the observed algal pigment reduction at higher irradiance as shown in Figure 6 and Figure 7 can provide further insights into the changes in effect. Some reports [41,42], also demonstrated that high C:N ratios were associated with reduced protein concentrations, decreases in chlorophyll-a, and the accumulation of carbon storage products. In addition, previous studies have reported a decrease in the chlorophyll-a concentration under low nitrogen conditions that hinder protein synthesis in many microalgae species. Satya et also observed a trend of an in-line increase in chlorophyll and phycocyanin content with the protein synthesis in *Spirulina fusiformis* culture, although the increasing trend that was observed with rising light intensity up to 10,000 lux (~81.9 W/m^2^) is in contrast with the observation in this experiment. This contrast observation is possibly due to different light availability and can be explained in terms of a light response curve model, as the light range up to 10,000 lux (~81.9 W/m^2^) in a laboratory culture is below the saturated point for the algal physiology [25], while in this outdoor experiment the average daily irradiance range was considerably higher, up to more than 25,000 lux (~204.9 W/m^2^) (Figure 2).

Maintaining a light condition that favors algal protein synthesis requires a strategy to optimize the outdoor algal culture for the purpose of pigment production. This light level is in line with what cyanobacterial needs in order to grow and synthesize proteins, as for enhancing the outdoor cyanobacterial culture productivity for any purposes, steps must be taken in controlling daylight at a range that protects cyanobacterial cells from sudden peaks of sunlight exposure.

## 6. Conclusions

This study reveals that there is a lethal hazard risk in a closed-column cyanobacterial culture set in a tropical place, which is related to heat accumulation. The risk can be diminished by utilizing shading and column size. Paranet roofing can enable external protection to enhance cyanobacterial culture resilience against oversaturated peak daylight. Resilience can also be achieved by encouraging culture temperature buffer capacity as well as the cyanobacterial culture self-density regulation through the column surface-to-volume ratio. The point of control to enhance outdoor cyanobacterial culture productivity is at the light level by favoring cyanobacterial cells photosynthetic in balance with the protein synthetic processes, which in this study was mostly achieved by a combination of a paranet roofing of 70% light reduction level and a column diameter of 12.74 cm which represented in PBRd-20.

## Figures and Tables

**Table 1:** Growth performance and biochemicals content of *A. platensis* in outdoor inclined plastic column culture.

	Shading Treatment			
	I-100	I-50	I-30	I-10
**PBRd-20**				
Culture Density (g/L)	0.86 ± 0.41	1.44 ± 0.44	1.29 ± 0.45	0.40 ± 0.09
Specific Growth Rate (%/day)	22.98 ± 12.42	22.28 ± 13.33	21.86 ± 12.32	19.31 ± 6.13
Biomass Productivity (mg/(L·day))	108.94 ± 52.04	149.03 ± 60.08	129.89 ± 61.29	42.08 ± 11.98
Protein (% DW)	51.74 ± 7.73	55.29 ± 10.43	54.10 ± 7.45	60.51 ± 6.32
Carbohydrate (% DW)	15.14 ± 6.89	12.83 ± 7.78	10.99 ± 4.88	7.55 ± 1.25
Lipid (% DW)	5.26 ± 1.69	6.56 ± 2.27	6.28 ± 1.41	5.67 ± 1.25
Ash (% DW)	13.84 ± 6.51	13.43 ± 5.93	16.95 ± 2.82	17.81 ± 2.70
Chlorophyll-a (% DW)	0.19 ± 0.04	0.23 ± 0.08	0.25 ± 0.07	0.27 ± 0.04
Phycocyanin (% DW)	8.39 ± 1.40	11.29 ± 1.98	13.18 ± 1.25	14.49 ± 1.98
**PBRd-50**				
Culture Density (g/L)	0.62 ± 0.17	0.57 ± 0.13	0.44 ± 0.09	0.18 ± 0.05
Specific Growth Rate (%/day)	19.96 ± 8.44	19.40 ± 9.71	19.01 ± 7.77	17.32 ± 5.87
Biomass Productivity (mg/(L·day))	63.71 ± 25.06	57.90 ± 25.21	44.78 ± 14.51	18.90 ± 7.16
Protein (% DW)	49.01 ± 7.39	58.56 ± 7.41	61.40 ± 5.86	66.73 ± 7.41
Carbohydrate (% DW)	12.31 ± 4.61	6.20 ± 3.31	7.50 ± 4.05	7.41 ± 3.30
Lipid (% DW)	5.31 ± 2.34	6.88 ± 1.77	6.55 ± 1.28	9.08 ± 2.27
Ash (% DW)	16.38 ± 2.83	15.48 ± 3.80	14.91 ± 3.69	9.07 ± 4.30
Chlorophyll-a (% DW)	0.18 ± 0.06	0.27 ± 0.08	0.29 ± 0.08	0.27 ± 0.05
Phycocyanin (% DW)	9.12 ± 2.34	13.17 ± 1.41	12.96 ± 1.11	14.91 ± 3.34

Remarks: I-100, I-50, I-30, and I-10 are the respective control treatment (without shading) means received of 50%, 30%, and 10% the transmitted light; DW is dry weight; PBRd-20 and PBRd-50 are photobioreactors which were 20 cm and 50 cm column width, giving the column a diameter of 12.74 cm and 31.85 cm, respectively, once fully filled with culture broth.

## Data Availability

The data presented in this study are available in the main article.
